# All in the Family: systematic reviews, rapid reviews, scoping reviews, realist reviews, and more

**DOI:** 10.1186/s13643-015-0163-7

**Published:** 2015-12-22

**Authors:** David Moher, Lesley Stewart, Paul Shekelle

**Affiliations:** Clinical Epidemiology Program, Ottawa Hospital Research Institute, School of Epidemiology, Public Health and Preventive Medicine, University of Ottawa, 501 Smyth Rd, Room L1288, Ottawa, K1H 8L6 ON Canada; Canadian EQUATOR Centre, Ottawa, Canada and The Ottawa Hospital, General Campus, 501 Smyth Rd, Room L1288, Ottawa, K1H 8L6 ON Canada; Centre for Reviews and Dissemination, University of York, York, YO10 5DD UK; Division of General Internal Medicine, West Los Angeles VA Medical Center, Los Angeles, California 90066 USA

Three years ago, we founded this journal focusing on publishing systematic reviews, systematic review protocols, and associated methodological development papers. Systematic reviews, which developed in the social sciences in the 1970s, began to gain rapid momentum during the 1990s in response to concerns by policymakers and clinicians about the scientific validity of the prevailing paradigm of traditional narrative reviews written by authoritative experts. What distinguished systematic reviews was the use of formal explicit methods, in other words pre-specification, of what exactly was the question to be answered, how evidence was searched for and assessed, and how it was synthesized in order to reach the conclusion. Importantly, these formal methods were described as part of the review itself in a Methods section. In turn, these methods themselves became the subject of hypothesis-testing studies, as investigators sought how best to search for evidence, assess studies for quality or risk of bias, determine under what conditions meta-analysis was justified, and how to best determine and characterize our confidence in the conclusions. The results of these hypothesis-testing studies led over time to improvements in the methods we use for systematic reviews. Thus, systematic reviews and this journal are part of an ever-evolving process whose foundations are rooted in the scientific method.

Today, new forms of reviews are appearing. These have emerged in response to policymakers and other stakeholders’ needs for information, sometimes emergently or urgently, for which the existing systematic review model does not quite fit. Hence, the rapid review, when time is of the essence; the scoping review, when what is needed is not detailed answers to specific questions but rather an overview of a broad field; the evidence map, a cousin of scoping reviews that commonly has a specific visual presentation of the evidence across a broad field; and the realist review, where the question of interest includes how and why complex social interventions work in certain situations, rather than assume they either do or do not work at all.

As Editors, we regularly get contacted by prospective authors asking whether realist reviews, or scoping reviews, etc., are “within the scope” for Systematic Reviews, and therefore we judge it an opportune time to state our position on this matter.

It is our view that all of these new forms of reviews are related to systematic reviews, similar to the way that different biological Species within the same Family are related to each other. We consider “systematic reviews” to be the Family and the different forms of reviews to be the different species. Just as in the biological classification, where the related organisms must share certain characteristics, so too must the different types of reviews share a common characteristic. In this case, what is shared is their foundation in the scientific method, with their methods articulated in advance in sufficient detail that the review can be reproduced by others. A further defining feature is that there are scholars devoted to improving the methods over time via hypothesis-testing studies.

*Systematic Reviews* has responded to these emerging techniques and already publishes papers reporting methods and findings from the systematic review family and has for example published an extremely popular series on rapid reviews. We would like to consolidate this position and invite submission of these new species of reviews, as long as they meet the qualifications just listed. We expect this process of evolution to continue and to refine the methods such that in 10 years’ time, there will appear new, as yet unimagined species of reviews—and perhaps some current species of review will become extinct. Thus, it is with the scientific method, which we believe is the foundation that unites the Family of systematic reviews.

